# Hepatitis E virus infection in Europe: surveillance and descriptive epidemiology of confirmed cases, 2005 to 2015

**DOI:** 10.2807/1560-7917.ES.2017.22.26.30561

**Published:** 2017-06-29

**Authors:** Esther J Aspinall, Elisabeth Couturier, Mirko Faber, Bengü Said, Samreen Ijaz, Lara Tavoschi, Johanna Takkinen, Cornelia Adlhoch, on behalf of the country experts

**Affiliations:** 1 NHS National Services Scotland, Glasgow, United Kingdom; 2 Glasgow Caledonian University, Glasgow, United Kingdom; 3 Sante Publique France, Saint-Maurice, France; 4 Robert Koch Institute, Berlin, Germany; 5 National Infection Service, Public Health England, London, United Kingdom; 6 European Centre for Disease Prevention and Control (ECDC), Stockholm, Sweden; 7 Country experts are listed at the end of the article

**Keywords:** Hepatitis E, Europe, surveillance, case definitions

## Abstract

Hepatitis E virus (HEV) is an under-recognised cause of acute hepatitis in high-income countries. The purpose of this study was to provide an overview of testing, diagnosis, surveillance activities, and data on confirmed cases in the European Union/European Economic Area (EU/EEA). A semi-structured survey was developed and sent to 31 EU/EEA countries in February 2016, 30 responded. Twenty of these countries reported that they have specific surveillance systems for HEV infection. Applied specific case definition for HEV infection varied widely across countries. The number of reported cases has increased from 514 cases per year in 2005 to 5,617 in 2015, with most infections being locally acquired. This increase could not be explained by additional countries implementing surveillance for HEV infections over time. Hospitalisations increased from less than 100 in 2005 to more than 1,100 in 2015 and 28 fatal cases were reported over the study period. EU/EEA countries are at different stages in their surveillance, testing schemes and policy response to the emergence of HEV infection in humans. The available data demonstrated a Europe-wide increase in cases. Standardised case definitions and testing policies would allow a better understanding of the epidemiology of HEV as an emerging cause of liver-related morbidity.

## Introduction

Hepatitis E virus (HEV) infection is one of the leading causes of acute viral hepatitis worldwide with four different genotypes (1–4) responsible for most human infections. HEV genotype 3 predominates in high-income countries, including those in Europe. Transmission of this genotype is usually zoonotic and has been linked to the consumption of pork products, and in some instances, shellfish [1-3]. Infection, which is most often asymptomatic, may cause an acute self-limiting hepatitis, with symptomatic infection more commonly reported among men older than 50 years of age [4,5]. Reports of chronic infection among immunocompromised people or those with pre-existing liver disease have been described [4].

There is evidence that HEV is an under-recognised pathogen in high-income countries, and that the incidence of confirmed cases has been steadily increasing over the last decade [6-10]. Although population studies have shown stable or decreasing seroprevalence rates [11,12], some countries have reported a consistently high seroprevalence and proportion of HEV-RNA-positive blood donors [13-16]. A systematic review conducted by the World Health Organization (WHO) reported HEV seroprevalence (as denoted by presence of IgG antibody) to range from 0.03% to 52% among the general population and blood donors in the WHO European Region, with the highest prevalence reported from studies in blood donors from France and the Netherlands [17]. Evidence of current HEV infection in up to 77% of patients presenting with symptomatic acute hepatitis suggests that the virus could be a considerable cause of liver morbidity in European countries [17]. However, the use of different serological test systems significantly influences the estimates of seroprevalence [18].

Europe-wide surveillance is not in place for hepatitis E, and the European Centre for Disease Prevention and Control (ECDC) has launched a number of activities that aim to better understand the current epidemiology, as well as the national monitoring systems for HEV infection within European Union/European Economic Area (EU/EEA) countries. A recent study involving HEV experts from 17 countries documented an increase in reported cases in nearly all EU/EEA countries that provided data, with France, Germany, England and Wales, and the Netherlands reporting more hepatitis E than hepatitis A notifications. The study also highlighted a lack of consistency in the reporting of cases, and wide variation in surveillance activity and case definitions [7]. The current study builds on this work to conduct a wider assessment of testing, diagnosis, surveillance activities and confirmed cases of HEV infection in the EU/EEA in order to inform the Europe-wide response to this emerging infection.

## Methods

A semi-structured survey of EU/EEA countries was conducted during 2015/16. The survey was divided into four sections (surveillance, testing and diagnosis, data on diagnosed cases, and transfusion-associated HEV infections) to allow separate country-level respondents for each topic area with a total of 24 open and closed questions [19]. A pilot survey was conducted at a meeting of the ECDC HEV expert group in December 2015, and a revised survey was subsequently circulated by email to nominated representatives of ECDC’s networks, the National Focal Points for Food- and Waterborne Diseases and Zoonoses as well as for Microbiology and Surveillance, in February 2016. Respondents were given three weeks to complete the survey (which could be completed electronically), and email and phone reminders were used to follow up with those who had not responded.

Returned survey data were manually extracted into Excel and double-checked. Any unclear responses were checked by email communication with the respective experts. Analyses were conducted in Excel and STATA version 13. The United Kingdom (UK) provided three separate responses (from England and Wales, Scotland, and Northern Ireland) which were considered as a single country response for all quantitative analyses and listed separately for qualitative analyses. A descriptive analysis was performed. The proportion of hospitalised cases was calculated using data exclusively from countries reporting hospital data, with the number of total cases as denominator. The Eurostat population database from 2015 was used as source of population denominator data [20].

## Results

All EU/EEA countries with the exception of Liechtenstein responded to the survey, giving a response rate of 30 out of 31. The data provided from countries that conduct surveillance was comprehensive, detailed and of high quality.

### Hepatitis E surveillance systems

Of 30 responding countries, 20 had specific surveillance systems for HEV infection. The remaining 10 countries had more generic viral hepatitis syndromic surveillance with no plans to develop any specific surveillance for HEV infection. Of the 20 EU/EEA countries with specific systems for HEV infection, 15 had national surveillance systems, three had laboratory surveillance through national reference laboratories with unknown coverage, one had blood service surveillance with unknown coverage, and one had a sentinel surveillance system covering approximately half of the population.

**Figure 1:**
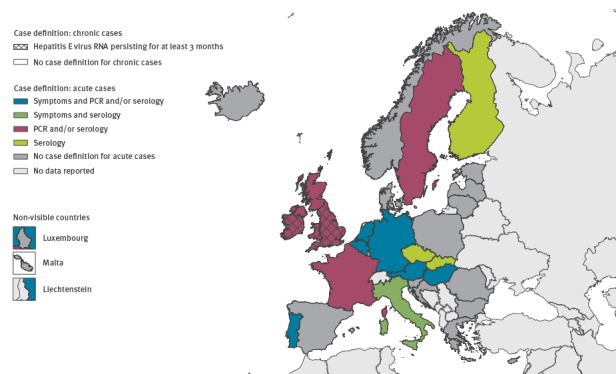
Type of applied case definition for confirmed cases of hepatitis E virus infection in 20 European Union/European Economic Area (EU/EEA) countries, 2015

PCR: polymerase chain reaction; RNA: ribonucleic acid.

Thirteen of 20 surveillance systems specific for HEV infection had a case definition for confirmed cases. There was wide variation in the type of case definition used: seven used laboratory and clinical definitions, and six used a laboratory definition only (Figure 1). Most surveillance systems collected demographic information as well as the date of notification and onset of disease, whereas morbidity and mortality data were collected less frequently (Table 1). Two countries’ surveillance systems (Ireland and the UK (England and Wales)) differentiated between acute and chronic HEV infection: acute infection was defined as both HEV-IgM-positive and IgG-positive, or HEV-RNA-positive, and chronic infection was defined as HEV RNA persisting for at least 3 months.

Administrative boundaries from EuroGraphics. Map produced on 8 June 2017.

**Table 1:** Variables collected by specific surveillance systems for hepatitis E virus infection in 20 European Union/European Economic Area (EU/EEA) countries, 2015

Countries collecting data on hepatitis E cases via their surveillance systems	Variables
**Majority (> 14 countries)**	Unique patient identifier, date of notification, source of notification, date of birth, sex, date of onset of disease
**Some (6–14 countries)**	Date of diagnosis, cluster link, occupation, pregnancy, clinical symptoms, travel, food history, contact with animals, hospitalisation, death
**Few (< 6 countries)**	Ethnicity, migration status, alcohol consumption, medication, immunosuppressive medication or condition, other medical conditions, past transfusion/transplantation

### Hepatitis E testing and diagnosis

Twenty-six countries conducted HEV testing in their own countries, with two countries sending samples abroad for testing and two reporting their testing availability as unknown. In 12 countries, there were less than five laboratories able to conduct diagnostic tests for HEV. Laboratories in 20 countries used more than one type of test (Table 2). For HEV antibody testing in acute cases, IgM ELISA was used in 21 countries and IgM Western Blot in 11. Of the 22 countries using any IgM antibody test, 20 also used IgG ELISA and nine IgG Western Blot for the detection of IgG antibodies. Nineteen countries reported that they were able to conduct HEV RNA testing; all used serum/plasma and 12 also used stool specimens. Seventeen countries reported that HEV sequencing was conducted for epidemiological investigations (15 countries), for research purposes (14 countries) or as routine practice (10 countries). Ten countries provided information on why a laboratory might conduct a test for HEV. Two countries had a laboratory policy on testing for HEV: (i) UK (Scotland), where some laboratories automatically test for HEV if alanine transaminase (ALT) was ≥ 100 U/L, and (ii) Ireland, where an HEV test is automatically conducted if a test for hepatitis A virus (HAV) is requested. The remaining countries reported that HEV testing was conducted only at the request of a clinician.

**Table 2:** Types of tests used to detect hepatitis E virus infection in 28 European Union/European Economic Area (EU/EEA) countries, 2015

Country	IgM ELISA			IgM Western blot			IgG ELISA			IgG Western blot			PCR Serum			PCR Stool			Other
	Acute	Chronic	Test^a^	Acute	Chronic	Test^a^	Acute	Chronic	Test^a^	Acute	Chronic	Test^a^	Acute	Chronic	Test^a^	Acute	Chronic	Test^a^	EM
Belgium	Y	N	Wantai	N	N	–	Y	N	Wantai	N	N	–	Y	Y	RealStar HEV RT-PCR, Altona	Y	–	In-house PCR	N
Bulgaria	Y	–	–	–	–	–	Y	–	–	–	–	–	–	–	–	–	–	–	–
Croatia	Y	N	recomWell HEV IgM, Mikrogen	Y	–	recomLine HEV IgG/IgM, Mikrogen	Y	–	recomWell HEV IgG, Mikrogen	Y	–	recomLine HEV IgG/IgM, Mikrogen	Y	–	[25,26]	N	–	–	N
Cyprus	Y	N	Radim no. KH3 IW	N	N	–	Y	N	Radim no. KH1 IW	N	N	–	N	N	–	N	–	–	N
Czech Republic	Y	N	–	N	N	–	Y	N	–	N	N	–	Y	N	–	Y	N	–	N
Denmark	Y	–	–	N		–	Y		–	N	–	–	Y		–	Y	–	–	N
Estonia	Y	N	recomWell HEV IgM, Mikrogen	Y	N	recomLine HEV IgG/IgM, Mikrogen	Y	N	recomWell HEV IgG Mikrogen	Y	N	recomLine HEV IgG/IgM, Mikrogen	Y	N	In-house PCR	N	N	–	N
Finland	Y	N	recomWell HEV IgM, Mikrogen	N	N	–	Y	N	recomWell HEV IgG, Mikrogen	N	N	–	Y	–	–	N	–	–	N
France	N	N	–	N	N	–	N	N	–	N	N	–	Y	Y	–	Y	Y	–	N
Germany	Y	N	recomWell HEV IgM, Mikrogen; HEV IgM, Euroimmun	Y	N	recomLine HEV IgG/IgM, Mikrogen	Y	N	recomWell HEV IgG, Mikrogen; HEV IgG, Euroimmun	Y	N	recomLine HEV IgG/IgM, Mikrogen	Y	Y	In-house PCR; RealStar HEV RT-PCR, Altona; Mikrogen; Ceeram; Roche	Y	Y	In-house PCR; RealStar HEV RT-PCR, Altona; Mikrogen; Ceeram; Roche	Y
Greece	N		–	N			N		–	N		–	N		–	N		–	N
Hungary	Y	N	Mikrogen; DiaPro	Y	N	Mikrogen	N	N	–	N	N	–	Y	Y	In-house PCR	Y	Y	In-house PCR	N
Iceland	N	N	–	N	N	–	N	N	–	N	N	–	N	N	–	N	N		N
Ireland	Y	N	Fortress	N	N	–	Y	N	Fortress	N	N	–	Y	Y	RealStar HEV RT-PCR, Altona	Y	N	RealStar HEV RT-PCR, Altona	N
Italy	Y	N	Wantai	N	N	–	Y	N	Wantai	N	N	–	Y	N	RealStar HEV RT-PCR, Altona	N	N		N
Latvia	Y	–	recomWell HEV IgM, Mikrogen; HEV IgM Kit, EIAgen Adaltis	N	–	–	Y	–	recomWell HEV IgG, Mikrogen; HEV IgG Kit, EIAgen Adaltis	N	–	–	Y	–	Geno-Sen’s HEV Real Time PCR Kit, Genome Diagnostics	N	–	–	N
Lithuania	N	–	–	Y	–	Mikrogen	N	–	–	Y		Mikrogen	N	N	–	N	N	–	N
Luxembourg	Y	N	Mikrogen	N	–	–	Y	N	Mikrogen	N	–	–	N	–	–	N	–	–	N
The Netherlands	N	N	–	Y	N	–	N	N	–	N	N	–	Y	Y	–	Y	Y	–	N
Norway	Y	Y	IgM, Wantai	N	N	–	Y	Y	IgG, Wantai	N	N	–	Y	Y	RealStar HEV RT-PCR, Altona	N	N	–	N
Poland	Y	–	–	Y		–	Y	–	–	Y	–	–	N	–	–	N	–	–	N
Portugal	Y	–	–	N		–	Y	–	–	N	–	–	Y	–	–	Y	–	–	N
Romania	N	N	–	N	N	–	N	N	–	N	N	–	N	N	–	N	N	–	N
Slovakia	Y	N	–	Y	N	–	Y	N	–	Y	N	–	–	–	–	–	–	–	–
Slovenia	N	Y	–	Y	Y	–	N	Y	–	Y	Y		Y	Y	–	Y	Y	–	–
Spain	Y	Y	DSI	Y	Y	Mikrogen	Y	Y	DSI	Y	Y	Mikrogen	Y	Y	In-house PCR	Y	Y	In-house PCR	N
Sweden	Y	Y	Mikrogen; DiaPro; Wantai	N	N	–	Y	Y	Mikrogen; DiaPro; Wantai	N	N	–	Y	Y	In-house PCR	Y	Y	In-house PCR	N
UK - E + W	Y	Y	Wantai; Mikrogen	N	N	–	Y	Y	Wantai; Mikrogen	N	N	–	Y	Y	In-house qPCR; commercial assays	Y	Y	In-house qPCR	N
UK - S	N	N	Mikrogen	N	N	–	N	N	Mikrogen	N	N	–	Y	Y	In-house PCR	Y	N	In-house PCR	N
UK - NI	N	–	–	N	–	–	N	–	–	N	–	–	N		–	N	–	–	N

–: not available; ELISA: enzyme-linked immunosorbent assay; EM: electron microscope; E + W: England and Wales; HEV: hepatitis E virus; IgG: Immunoglobulin G; IgM: Immunoglobulin M; N: no; NI: Northern Ireland; PCR: polymerase chain reaction; qPCR: quantitative polymerase chain reaction; RNA: ribonucleic acid; RT-PCR: reverse transcriptase-polymerase chain reaction; S: Scotland; Y: yes.

^a^ Assay type and brand name are separated by comma, brand names are separated by semicolon. Brand names: EIAgen Adaltis (Guidonia Montecelio, Italy); Altona diagnostics (Hamburg, Germany); Ceeram (La Chapelle sur Erdre, France); DiaPro Diagnostic BioProbes (Sesto San Giovanni, Italy); DSI Diagnostic System Italy (Saronno, Italy); Euroimmun (Lübeck, Germany); Fortress diagnostics (Antrim, the United Kingdom); Genome Diagnostics (Nijmegen, the Netherlands); Mikrogen (Neuried, Germany); Radim (Rome, Italy); Roche (Penzberg, Germany); Wantai (Beijing, China).

### Confirmed cases of hepatitis E virus infection

**Figure 2:**
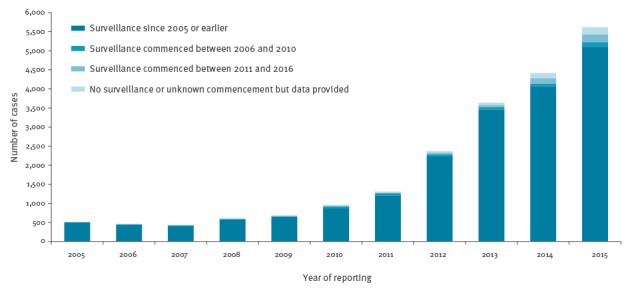
Reported number of cases of hepatitis E virus infection by year of notification and year of surveillance commencement, 22 European Union/European Economic Area (EU/EEA) countries, 2005–2015^a^

^a^ Data available for the following countries (year of commencement of HEV surveillance/year since cases reported during study period 2005–2015): Austria (1980/2012), Belgium (2010/2010), Bulgaria (no surveillance/2015), Croatia (2009/2009), Cyprus (no surveillance/2009), Czech Republic (1996/2005), Estonia (1997/2005), Finland (1995/2005), France (2002/2005), Germany (2001/2005), Hungary (1993/2005), Italy (2007/2007), Latvia (commencement unknown/2007), the Netherlands (2012/2012), Norway (no surveillance/2005), Poland (no surveillance/2014), Portugal (commencement unknown/2014), Slovakia (2007/2005), Slovenia (1995/2005), Spain (commencement unknown/2006), Sweden (1993/2005), and UK (England and Wales (2003/2005), Scotland (2000/2005) and Northern Ireland (unknown/2005).

Twenty-two countries covering 469 million people (i.e. 91% of the EU/EEA population of 514 million people) provided data on confirmed cases of hepatitis E (Figure 2). The total number of reported cases increased from 514 cases in 2005 to 5,617 cases in 2015, with 21,018 cases overall during the 11-year period studied (Figure 2). A total of 93% (19,531/21,018) of cases were accounted for by countries that have had hepatitis E-specific surveillance since at least 2005, and 80% (16,810/21,018) of cases were reported from just three countries (Germany, France, and the UK), which comprise 41% of the EU/EEA population. In 16 countries providing data on the age of reported cases, the overall proportion of those aged more than 50 years increased from 30–47% during 2005–2008 to 60–61% during 2013–2015. In 17 countries providing data on the sex of reported cases, the proportion of male cases remained stable over 2005–2015, ranging from 61% in 2005 and 2015 to 69% in 2006.

### Severity associated with hepatitis E virus infection

**Figure 3:**
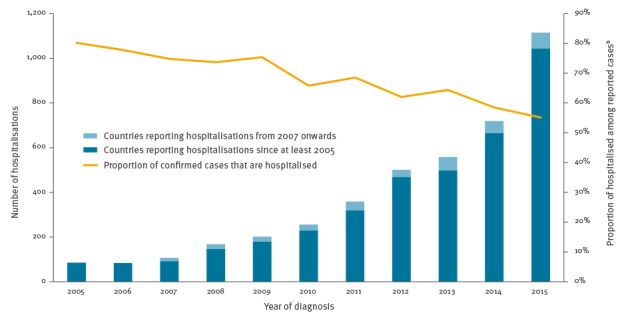
Number and proportion of hospitalisations among reported cases of hepatitis E virus infection, 14 European Union/European Economic Area (EU/EEA) countries, 2005–2015^a^

^a^ Data available for (year of first report): Austria (2012), Belgium (2010), Croatia (2012), Czech Republic (2005), Estonia (2012), Germany (2005), Hungary (2005), Italy (2007), Latvia (2007), Poland (2014), Portugal (2015), Slovakia (2005), Slovenia (2005) and the UK (Northern Ireland) (2013).

^b^ Proportion of hospitalisations only included denominator data from countries reporting both number of reported cases and number of hospitalised cases.

Data from 14 countries reporting on the total number of hospitalisations related to HEV infection showed an increase from 85 hospitalised cases in 2005 to 1,115 in 2015 (Figure 3). Most of this increase could be accounted for by four of the five countries that have conducted reporting on hospitalisation status since at least 2005. Over the same time period, the proportion of cases being hospitalised has decreased from 80% (85/106 cases) to 55% (1,115/2,023 cases). Twelve countries provided data on deaths associated with HEV infection, with five countries recording these data since 2005. In total, 28 fatal cases were reported by five countries (Austria, Czech Republic, Germany, Hungary and Italy), increasing from 0–2 cases per year during 2005–2011 to 4–8 cases per year during 2012–2015.

### Travel history of diagnosed cases

**Figure 4:**
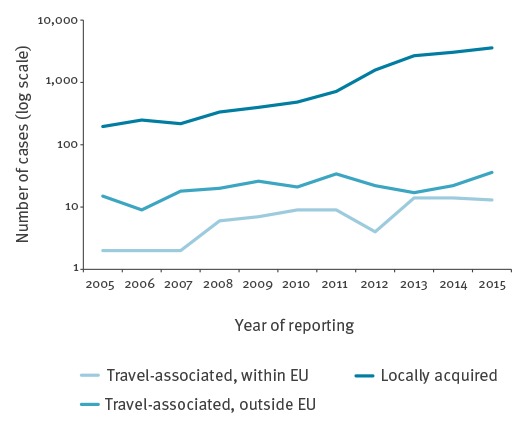
Reported cases of hepatitis E virus infection by year of notification and travel history, 15 European Union/European Economic Area (EU/EEA) countries, 2005–2015^a^

^a^ Data available for: Austria, Croatia, Czech Republic, Estonia, France, Hungary, Italy, Latvia, Poland, Portugal, Slovakia, Slovenia, Spain, Sweden and the UK (England, Northern Ireland and Wales).

Fifteen countries provided data on travel history. Of hepatitis E cases reported in these countries during the period 2005–2015, 87% (13,511/15,525) were autochthonous (defined as locally acquired within the reporting country). The proportion of known autochthonous cases increased from 45–73% per year during 2006–2011 to 89–97% per year during 2012–2015. This suggests that the considerable increase in confirmed cases observed after 2011 (Figures 2 and 4) can largely be accounted for by locally acquired infection. A very small number of cases per year (9–36 cases) were known to be associated with travel outside of the EU/EEA.

## Discussion

This study assessed testing policy and practice, laboratory diagnosis, and surveillance systems and activities for HEV infection in 31 EU/EEA countries. Thirty of the countries responded to the survey, providing the most comprehensive picture to date of HEV as an emerging infection among humans in Europe. The 10-fold increase in the number of cases reported between 2005 and 2015, more than 21,000 confirmed cases overall and a total of 28 deaths, underline the relevance and emerging nature of this zoonotic infection in Europe.

The survey findings demonstrate a varied response to hepatitis E across EU/EEA countries; while two thirds of countries have established surveillance systems, a third of countries have no specific surveillance and no plans to develop any. For those with existing surveillance, nearly one third do not apply a case definition for HEV infection nationally. Only 19 countries reported that they were able to conduct HEV RNA testing in-country, although there may be testing within hospitals or private settings that the national public health institutes or survey respondents are unaware of. In most countries, it was not possible to ascertain the criteria for referral or for HEV test requests. While there may be an increasing awareness of locally acquired HEV infections, there is still concern that within parts of the clinical community, HEV is not considered as a possible cause of hepatitis unless there is a recent history of travel. It is therefore likely that the infection remains under-diagnosed. There also appears to be a gap in knowledge around the testing protocols used in outpatient and hospital settings within countries, which should be investigated further to understand when, who, and why patients are tested for HEV.

Twenty-two countries (comprising more than 90% of the total EU/EEA population) were able to provide data on confirmed cases for at least some of the period 2005–2015. These data demonstrate that the number of reported cases has been increasing year-on-year, with a more than threefold increase between 2011 and 2015. The majority of cases were reported by three countries (the UK, France and Germany) that have had surveillance systems in place since at least 2005. A small number of countries reported changes to surveillance systems during the study period; for example, Belgium created a national reference centre for HEV in 2011 and Portugal commenced web-based notification in 2014. However, there were no changes that could have contributed substantially to the increase in the number of confirmed cases. A greater awareness of and increased testing for HEV, particularly of patients presenting with acute hepatitis by general practitioners in outpatient settings, is likely to be one of the contributing factors e.g. through the inclusion of HEV in some standard diagnostic protocols. The rise in the number of symptomatic cases might also be related to the switch in virus subtypes that has been observed in some EU/EEA countries [7,21].

The concurrent increase in number of hospitalisations (a measure of increased HEV infection severity) and gradual decrease in proportion of total cases hospitalised between 2005 and 2015 may suggest better outpatient diagnosis and an increase in the sensitivity of the diagnostic system, i.e. a change in testing practice from hospitals to community settings. For cases where clinical information was recorded, every second case was admitted to hospital and there were two to eight deaths associated with HEV infection annually during 2011–2015. These figures appear high for a disease that has previously been considered to be a mild self-limiting infection [22]. However, a testing bias and overestimation of the hospitalisation rate can be assumed because of a higher likelihood that cases of severe liver disease or other comorbidities are tested for HEV. Notably, only four countries were able to provide data on morbidity status since 2005 and only five were able to provide data on case fatality since 2005, although nearly half of the EU/EEA countries indicated that they are able to collect this information as of 2015. More data are needed to address the lack of knowledge in this area.

As our results show, the number and proportion of autochthonous HEV infections has been increasing since 2005. Only a very small number of cases were known to be related to travel outside of the EU/EEA. Although genotype data were not requested from countries, it is known that autochthonous cases tend to be due to HEV genotype 3, the same genotype present in European pigs and consumed pork products; travel-related cases would be expected to be infected with genotype 1 or 4 [2-5]. Lately, there appears to have been a change in circulating subtypes in Europe. For example, the number of subtype 3c recently exceeded the numbers of subtypes 3e, f and g in humans in England and Wales [21]. Such changes may be contributing to the emergence of HEV as an important infection in humans. Changes in the pattern of food preparation and consumption may also be implicated. A case–control study in England and Wales identified pork sausages and ham purchased in supermarkets as possible sources of infection, raising concerns about whether current practice in preparing these products is sufficient to prevent HEV infection [23]. More studies among humans, animal populations and on food are essential to informing on possible sources of HEV infection.

The main limitations of this study are the variation in the denominator data for reported cases of hepatitis E (most countries were only able to provide a subset of demographic information or a limited number of years of data) and the merging of data on reported cases of hepatitis E despite the different case definitions and testing approaches used by EU/EEA countries. These limitations have in part been addressed by showing data by year of surveillance introduction and by limiting the denominator to cases where the relevant information was known. The varying number of countries providing data inconsistently over the study period prompted us not to perform any trend analysis to avoid further introduction of bias. In the future these limitations could be addressed by standardisation of surveillance systems for HEV infection, harmonisation of the case definition as well as testing algorithms across EU/EEA countries. This would enable better assessment of the burden of disease due to HEV in Europe and facilitate a better understanding of the actual number of performed tests or population coverage as a denominator, thus avoiding diagnostic and surveillance artefacts.

In summary, EU/EEA countries are at different stages in their surveillance and testing capacity for HEV, and there is no standardised European case definition. For the time period 2005–2015, at least 22 countries were able to report on cases infected with HEV, either through formal surveillance or existing systems of laboratory notifications. WHO’s global health sector strategy on viral hepatitis asks countries to establish surveillance for viral hepatitis, particularly in blood donors [24]. Our study describes the availability of general hepatitis E surveillance in most EU/EEA countries. These data demonstrate a Europe-wide increase in reported cases, as well as a high number of reported HEV-related hospitalisations and a total of 28 deaths, between 2005 and 2015. They also provide a better understanding of the epidemiology and burden of HEV as an emerging cause of liver-related morbidity in EU/EEA countries.

## Notes

**Country experts:** Veronik Hutse, National Reference Centre for Hepatitis E Virus in Belgium, Scientific Institute of Public Health, Brussels, Belgium Vanessa Suin, National Reference Centre for Hepatitis E Virus in Belgium, Scientific Institute of Public Health, Brussels, Belgium Sanja Kurečić Filipović, Croatian Institute of Public Health, Zagreb, Croatia Maria Koliou, Ministry of Health, Nicosia, Cyprus Steen Ethelberg, Statens Serum Institut, Copenhagen, Denmark Jevgenia Epstein, Health Board, Tallinn, Estonia Ruska Rimhanen-Finne, National Institute for Health and Welfare (THL), Helsinki, Finland Mia Kontio, National Institute for Health and Welfare (THL), Helsinki, Finland Kassiani Mellou, Hellenic Centre for Disease Prevention and Control, Athens, Greece Ágnes Fehér, Ministry of Human Capacities, Budapest, Hungary Lelia Thornton, Health Service Executive, Health Protection Surveillance Centre, Dublin, Ireland Maria Elena Tosti, National Institute of Health (Istituto Superiore di Sanità - ISS), Rome, Italy Anna Rita Ciccaglione, National Institute of Health (Istituto Superiore di Sanità - ISS), Rome, Italy Rita Korotinska, Centre for Disease Prevention and Control, Riga, Latvia Wilfrid van Pelt, National Institute for Public Health and the Environment, Bilthoven, the Netherlands Paul Cabrey, Public Health Agency, Belfast, the United Kingdom Line Vold, Norwegian Institute of Public Health, Oslo, Norway Heidi Lange, Norwegian Institute of Public Health, Oslo, Norway Cátia Sousa Pinto, Directorate-General of Health, Lisbon, Portugal Rita de Sousa, National Institute of Health Doutor Ricardo Jorge (INSA), Lisbon, Portugal Małgorzata Sadkowska-Todys, National Institute of Public Health, Warsaw, Poland Adriana Pistol, National Institute of Public Health, Bucharest, Romania Denisa Janță, National Institute of Public Health, Bucharest, Romania Alison Smith-Palmer, Health Protection Scotland, NHS National Services Scotland, Glasgow, the United Kingdom Ana Avellon, Spanish National Centre of Microbiology, Carlos III Institute of Health, Madrid, Spain Helena Hudecová, Public Health Authority of the Slovak Republic, Bratislava, Slovakia Jan Mikas, Public Health Authority of the Slovak Republic, Bratislava, Slovakia Eva Grilic, National institute of Public Health (Nacionalni inštitut za javno zdravje - NIJZ), Ljubljana, Slovenia Lena Sundqvist, Public Health Agency of Sweden, Stockholm, Sweden

## References

[1] Said B Ijaz S Kafatos G Booth L Thomas HL Walsh A Hepatitis E outbreak on cruise ship. Emerg Infect Dis. 2009;15(11):1738-44. 10.3201/eid1511.091094 19891860 PMC2857258

[2] Colson P Borentain P Queyriaux B Kaba M Moal V Gallian P Pig liver sausage as a source of hepatitis E virus transmission to humans. J Infect Dis. 2010;202(6):825-34. 10.1086/655898 20695796

[3] Riveiro-Barciela M Mínguez B Gironés R Rodriguez-Frías F Quer J Buti M Phylogenetic demonstration of hepatitis E infection transmitted by pork meat ingestion. J Clin Gastroenterol. 2015;49(2):165-8. 10.1097/MCG.0000000000000113 24637729

[4] Nelson KE Kmush B Labrique AB The epidemiology of hepatitis E virus infections in developed countries and among immunocompromised patients. Expert Rev Anti Infect Ther. 2011;9(12):1133-48. 10.1586/eri.11.138 22114964

[5] Lapa D Capobianchi MR Garbuglia AR Epidemiology of Hepatitis E Virus in European Countries. Int J Mol Sci. 2015;16(10):25711-43. 10.3390/ijms161025711 26516843 PMC4632823

[6] Koot H Hogema BM Koot M Molier M Zaaijer HL Frequent hepatitis E in the Netherlands without traveling or immunosuppression. J Clin Virol. 2015;62:38-40. 10.1016/j.jcv.2014.11.020 25542468

[7] Adlhoch C Avellon A Baylis SA Ciccaglione AR Couturier E de Sousa R Hepatitis E virus: Assessment of the epidemiological situation in humans in Europe, 2014/15. J Clin Virol. 2016;82:9-16. 10.1016/j.jcv.2016.06.010 27393938

[8] Ijaz S Vyse AJ Morgan D Pebody RG Tedder RS Brown D Indigenous hepatitis E virus infection in England: more common than it seems. J Clin Virol. 2009;44(4):272-6. 10.1016/j.jcv.2009.01.005 19217345

[9] Pischke S Behrendt P Bock CT Jilg W Manns MP Wedemeyer H Hepatitis E in Germany--an under-reported infectious disease. Dtsch Arztebl Int. 2014;111(35-36):577-83. 25249359 10.3238/arztebl.2014.0577 PMC4174681

[10] Chalupa P Vasickova P Pavlik I Holub M Endemic hepatitis E in the Czech Republic. Clin Infect Dis. 2014;58(4):509-16. 10.1093/cid/cit782 24280093

[11] Holm DK Moessner BK Engle RE Zaaijer HL Georgsen J Purcell RH Declining prevalence of hepatitis E antibodies among Danish blood donors. Transfusion. 2015;55(7):1662-7. 10.1111/trf.13028 25819381

[12] Wenzel JJ Sichler M Schemmerer M Behrens G Leitzmann MF Jilg W Decline in hepatitis E virus antibody prevalence in southeastern Germany, 1996-2011. Hepatology. 2014;60(4):1180-6. 10.1002/hep.27244 24912687

[13] Hogema BM Molier M Slot E Zaaijer HL Past and present of hepatitis E in the Netherlands. Transfusion. 2014;54(12):3092-6. 10.1111/trf.12733 24889277 PMC4280434

[14] Tedder RS Tettmar KI Brailsford SR Said B Ushiro-Lumb I Kitchen A Virology, serology, and demography of hepatitis E viremic blood donors in South East England. Transfusion. 2016;56(6pt2):1529-36. 10.1111/trf.13498 26841005

[15] Mansuy JM Legrand-Abravanel F Calot JP Peron JM Alric L Agudo S High prevalence of anti-hepatitis E virus antibodies in blood donors from South West France. J Med Virol. 2008;80(2):289-93. 10.1002/jmv.21056 18098159

[16] Lucarelli C Spada E Taliani G Chionne P Madonna E Marcantonio C High prevalence of anti-hepatitis E virus antibodies among blood donors in central Italy, February to March 2014. Euro Surveill. 2016;21(30):30299. 10.2807/1560-7917.ES.2016.21.30.30299 27494608

[17] World Health Organization (WHO). A Systematic Review on Hepatitis E Virus Globally. Geneva: WHO; 2014. Available from: http://www.who.int/immunization/sage/meetings/2014/october/7_summary_HEV_systematic_review.pdf

[18] Hartl J Otto B Madden RG Webb G Woolson KL Kriston L Hepatitis E Seroprevalence in Europe: A Meta-Analysis. Viruses. 2016;8(8):211. 10.3390/v8080211 27509518 PMC4997573

[19] European Centre for Disease Prevention and Control (ECDC) Hepatitis E in the EU/EEA, 2005–2015. Stockholm: ECDC; Forthcoming.

[20] Eurostat. Population (Demography, Migration and Projections), Population data, Database, 2015. Luxembourg: Eurostat. [Accessed 7 Oct 2016]. Available from: http://ec.europa.eu/eurostat/web/population-demography-migration-projections/population-data/database

[21] Ijaz S Said B Boxall E Smit E Morgan D Tedder RS Indigenous hepatitis E in England and Wales from 2003 to 2012: evidence of an emerging novel phylotype of viruses. J Infect Dis. 2014;209(8):1212-8. 10.1093/infdis/jit652 24273173

[22] Sayed IM Vercouter AS Abdelwahab SF Vercauteren K Meuleman P Is hepatitis E virus an emerging problem in industrialized countries? Hepatology. 2015;62(6):1883-92. 10.1002/hep.27990 26175182

[23] Said B Ijaz S Chand MA Kafatos G Tedder R Morgan D Hepatitis E virus in England and Wales: indigenous infection is associated with the consumption of processed pork products. Epidemiol Infect. 2014;142(7):1467-75. 10.1017/S0950268813002318 24054519 PMC9151183

[24] World Health Organization (WHO). Global health sector strategy on viral hepatitis 2016–2021. Geneva: WHO. [Accessed 8 Jun 2017]. Available from: http://apps.who.int/iris/bitstream/10665/246177/1/WHO-HIV-2016.06-eng.pdf?ua=1

[25] van der Poel WHM Pavio N van der Goot J van Es M Martin M Engel B Development and validation of a genotype 3 recombinant protein-based immunoassay for hepatitis E virus serology in swine. Braz J Med Biol Res. 2014;47(4):334-9. 10.1590/1414-431X20133249 24676472 PMC4075298

[26] Jothikumar N Cromeans TL Robertson BH Meng XJ Hill VR A broadly reactive one-step real-time RT-PCR assay for rapid and sensitive detection of hepatitis E virus. J Virol Methods. 2006;131(1):65-71. 10.1016/j.jviromet.2005.07.004 16125257

